# The complete chloroplast genome of *Hevea camargoana*

**DOI:** 10.1080/23802359.2019.1710605

**Published:** 2020-01-14

**Authors:** Ying-Feng Niu, Yan-Shi Hu, Cheng Zheng, Zi-Yan Liu, Jin Liu

**Affiliations:** a Yunnan Institute of Tropical Crops, Xishuangbanna, China;; b Rubber Research Institute, Chinese Academy of Tropical Agriculture Science, Danzhou, China;; c Kunming Institute of Botany, Chinese Academy of Sciences, Kunming, China

**Keywords:** *Hevea camargoana*, chloroplast genome, phylogenetic

## Abstract

*Hevea camargoana* is a natural latex producing tropical plant and a close relative of *H. brasiliensis*, the primary commercial source of natural rubber. This study sequenced and analyzed the chloroplast genome of *H. camargoana*. The circular chloroplast genome of *H. camargoana* contains 161,291 bp with a GC content of 35.72%. This region contains two inverted repeat regions (26,819 bp), a large single-copy region (89,281 bp), and a small single-copy (18,372 bp) region in the complete chloroplast genome. A total of 134 genes were annotated, including 86 protein-coding genes, 36 transfer RNA genes, 8 ribosomal RNA genes, and 4 pseudogenes. The results showed that *H. camargoana* and *H. brasiliensis* were closely related, suggesting that *H. camargoana* may be used for the future variety improvement of rubber trees.

*Hevea camargoana* is a tropical plant that produces natural latex, native to the Amazon Basin and belongs to the family Euphorbiaceae. It is a close relative of *H. brasiliensis*, which is the primary commercial source for high-quality natural rubber (Rahman et al. 2013) and accounts for more than 98% of the total production worldwide (Pootakham et al. 2017). The genus *Hevea* contains 11 species (Gonçalves et al. 1990), most of which are diploid with 36 chromosomes (Lau et al. 2016). In addition to *H. brasiliensis* and *H. camargoana*, the other nine species are *H. bethamiana*, *H. guianensis*, *H. microphylla*, *H. pauciflora*, *H. rigidifolia*, *H. spruceana*, *H. paludosa*, *H. nitida*, and *H. camporum* (Priyadarshan and Goncalves 2002). The species *H. camargoana* has two specific characteristics: it is a dwarf plant with small leaves and it can be hybridized with *H. brasiliensis*. Therefore, it is a very important germplasm resource for the breeding dwarf and wind-resistant rubber trees, especially given the problem of a narrow genetic basis for utilizing its breeding potential (Tang et al. 2016).

The chloroplast is a plant organelle that contains its own genome with genes coding transcription and translation machinery as well as components of the photosynthetic complex (Tangphatsornruang et al. 2011). Sequencing information of the chloroplast is important for genetic improvement and toward an understanding of biological mechanisms of the plants (Shearman et al. 2014). Furthermore, the chloroplast sequences have often been used to study phylogenetic relationships between plants (Tangphatsornruang et al. 2010; Liu et al. 2018).

In this study, the chloroplast genome of *H. camargoana* has been sequenced and analyzed. Young leaves of *H. camargoana* were collected from The Rubber Tree Germplasm Resource Nursery of the Chinese Academy of Tropical Agriculture Science (N 19°34′31.53″and E 109°31′17.97″). The genomic DNA was isolated from the leaves using the Rapid Plant Genomic DNA Isolation Kit (Sangon Biotech Shanghai Co. Ltd., China). The DNA was stored in an ultra-low temperature specimen library at the Yunnan Institute of Tropical Crops (specimen accession number: YITC-2019-FZ-E-103). DNA was sequenced using the Illumina HiSeq 2000 (http://www.illumina.com, San Diego, CA, USA). The chloroplast genome of *H. camargoana* was assembled by CLC Genomics Workbench v3.6 (http://www.clcbio.com) and annotated by DOGMA (Wyman et al. 2004). The complete sequence and annotation results were submitted to GenBank, under the accession number MN781109.

The circular chloroplast genome of *H. camargoana* consists of 161,291 bp with a GC content of 35.72%, including 51,560 bp of A (31.97%), 52,117 bp of T (32.31%), 28,912 bp of G (17.93%), and 28,702 bp of C (17.80%). The complete chloroplast genome contains two inverted repeat regions (IRs, 26,819 bp), a large single-copy region (LSC, 89,281 bp), and a small single-copy (SSC, 18,372 bp) region. A total of 134 genes were annotated, including 86 protein-coding genes, 36 transfer RNA (tRNA) genes, 8 ribosomal RNA (rRNA) genes, and 4 pseudo genes.

Phylogenetic analyses (Figure 1) of *H. camargoana* and 17 other species (six species of the Euphorbiaceae family, five species of the Salicaceae family, five species of the Passifloraceae family, and *Betula platyphylla*, which belongs to the Betulaceae family and was used as outgroup) were conducted by MUSCLE v.3.8.31 (http://www.drive5.com/muscle/) (Edgar 2004). A phylogenetic tree was built by RAxML8.1.5 (https://sco.h-its.org/exelixis/web/software/raxml/index.html) (Stamatakis 2006), with a bootstrap value of 1000. The results showed that *H. camargoana* and *H. brasiliensis* were closely related, suggesting that *H. camargoana* may be used to improve the future variety of rubber trees.

**Figure 1.**
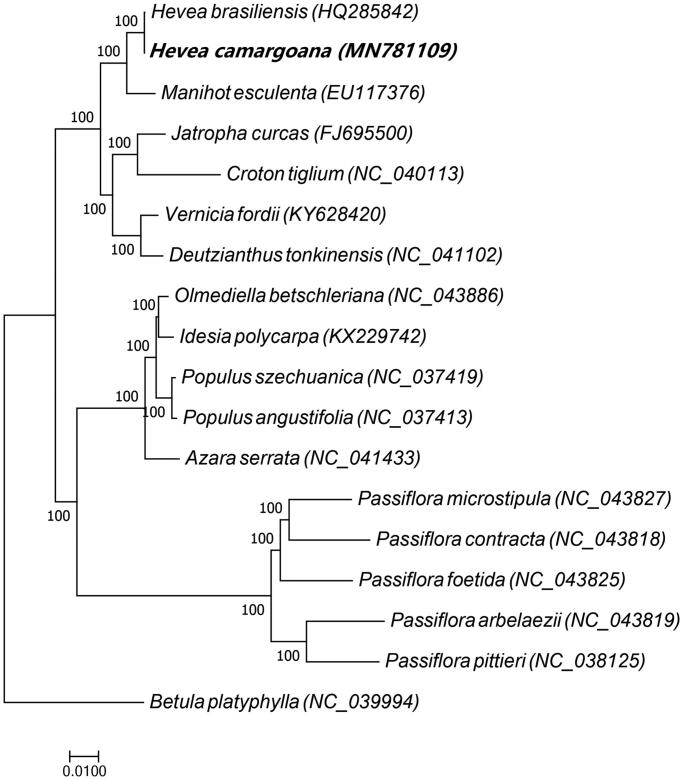
Maximum likelihood phylogenetic tree of *Hevea camargoana* and 17 other species (six species of the Euphorbiaceae family, five species of the Salicaceae family, five species of the Passifloraceae family, and *Betula platyphylla*, which belongs to the Betulaceae family and was used as outgroup). The bootstrap value was set to 1000. The species and chloroplast genome accession numbers for tree construction are: *H. camargoana* (MN781109), *H. brasiliensis* (HQ285842), *Manihot esculenta* (EU117376), *Jatropha curcas* (FJ695500), *Croton tiglium* (NC_040113), *Vernicia fordii* (KY628420), *Deutzianthus tonkinensis* (NC_041102), *Idesia polycarpa* (KX229742), *Olmediella betschleriana* (NC_043886), *Populus angustifolia* (NC_037413), *Populus szechuanica* (NC_037419), *Azara serrata* (NC_041433), *Passiflora contracta* (NC_043818), *Passiflora microstipula* (NC_043827), *Passiflora foetida* (NC_043825), *Passiflora pittieri* (NC_038125), *Passiflora arbelaezii* (NC_043819), and *Betula platyphylla* (NC_039994).
